# Aberrantly methylated-differentially genes and pathways among Iranian patients with colorectal cancer

**DOI:** 10.1186/s12935-021-02053-0

**Published:** 2021-07-03

**Authors:** Mahla Ghorbani, Marjan Azghandi, Mohammad Amin Kerachian

**Affiliations:** 1 Department of Biology, Faculty of Sciences, Mashhad Branch, Islamic Azad University, Mashhad, Iran; 2 Cancer Genetics Research Unit, Reza Radiotherapy and Oncology Center, Mashhad, Iran; 3 Department of Animal Science, Faculty of Agriculture, Ferdowsi University of Mashhad, Mashhad, Iran; 4 Medical Genetics Research Center, Mashhad University of Medical Sciences, Mashhad, Iran; 5 Department of Medical Genetics, Faculty of Medicine, Mashhad University of Medical Sciences, Mashhad, Iran

**Keywords:** Diagnostic, Colon adenocarcinoma, Epigenetic, In silico, Methylation

## Abstract

**Background:**

Methylation plays an important role in colorectal cancer (CRC) pathogenesis. The goal of this study was to identify aberrantly differentially methylated genes (DMGs) and pathways through bioinformatics analysis among Iranian CRC patients using Methylation Next Generation Sequencing.

**Methods:**

This study has integrated results of SureSelectXT Methyl-Seq Target with the potential key candidate genes and pathways in CRC. Six CRC and six samples of normal colon were integrated and deeply analyzed. In addition to this gene methylation profiling, several other gene methylation profiling datasets were obtained from Gene Expression Omnibus (GEO) and TCGA datasets. DMGs were sorted and candidate genes and enrichment pathways were analyzed. DMGs-associated protein–protein interaction network (PPI) was constructed based on the STRING online database.

**Results:**

Totally, 320 genes were detected as common genes between our patients and selected GEO and TCGA datasets from the Agilent SureSelect analysis with selecting criteria of p-value < 0.05 and FC ≥ 1.5. DMGs were identified from hyper-DMGs PPI network complex and 10 KEGG pathways were identified. The most important modules were extracted from MCODE, as most of the corresponding genes were involved in cellular process and protein binding.

**Conclusions:**

Hub genes including *WNT2*, *SFRP2*, *ZNF726* and *BMP2* were suggested as potentially diagnostic and therapeutic targets for CRC.

**Supplementary Information:**

The online version contains supplementary material available at 10.1186/s12935-021-02053-0.

## Introduction

Colorectal cancer (CRC) is one of the most common malignancies, which estimated more than 1.9 million new cases in developed countries in 2020. Overall, colorectal ranks third in terms of incidence, but second in terms of mortality [1]. In recent years, young Iranians show a rising trend of CRC scenarios probably due to their modern lifestyle and the young age structure of the country [2]. Despite detailed research into the mechanisms of CRC formation and growth, the causes of CRC are still unknown. Many factors, such as gene mutations, cellular and environmental factors, are linked to the development and growth of colon cancer [3]. Due to the high morbidity and mortality of CRC, it is critically necessary and strongly demanded to establish the causes and underlying molecular mechanisms for the discovery of molecular biomarkers for early detection, prevention and personalized therapy [4].

Epigenetics is defined as inherited variations of gene expression that is not regulated by changes in the DNA sequence. By altering the expression of several controlling genes, especially tumor suppressor genes, abnormal methylation can affect their functions and contribute to the development of various CRC processes [5]. While numerous studies have shown that several genes in CRC have abnormal DNA hyper- or hypo- methylation, the general profile and pathways of the interaction network are still unknown.[6].

Over the last few years, a number of methodologies have been established that rely on the use of bisulfide-converted DNA. Such techniques have not only been used to identify changes in gene or allele-DNA methylation but have also been modified for genome-DNA methylation analysis and are capable of delivering single-resolution DNA methylation information [7–10]. Epigenetics was one of the first molecular areas to benefit from next-generation sequencing (NGS) methodologies, offering a detailed and objective view of epigenome, and also freeing researchers from content-limited microarray platforms [11]. NGS technology has opened a new era in epigenomic science, especially in the area of DNA methylation. It is now possible to identify methylation status on a global scale with a single-base resolution by massively parallel sequencing [8]. The complete methylome's characterization, as well as its complex modifications, may be used as a precise diagnostic, prognostic, and predictive tool. Thus, detecting methylated-differentially expressed genes and learning more about their properties could be very helpful to discover the molecular mechanism and also the pathogenesis of CRC.

However, fewer investigations have narrowed the numbers of overlapping gene profiling to recognize the main genes and pathways involved in multiple cellular processes and biological functions. Now, by means of advanced bioinformatics analysis, more robust and precise screening results could be achieved by overlapping the related datasets.

To the best of our knowledge, there is no published research exploring molecular high-throughput methylation approach study in Iranian CRC patients except from our research team.

In the current study, we obtained Agilent SureSelect data, from our previous study [12]. Several other gene methylation profiling datasets were derived from Gene Expression Omnibus (GEO) and TCGA datasets in addition to this gene methylation profiling dataset. We filtered differentially methylated genes (DMGs), and consequently, developed Gene Ontology and pathway enrichment analysis for screening DMGs with DAVID, KEGG PATHWAY (available online: http://www.genome.jp/kegg), Reactome (available online: http://www.reactome.org), BioCyc (available online: http://biocyc.org), Panther (available online: http://www.pantherdb.org) [13], NHGRI and Gene Ontology online website for developing integration of DMGs protein–protein interaction (PPI) network (available online: http://string-db.org) and conducted modular analysis to identify hub genes in CRC (Additional file 1: Figure S1). Identifying DMGs and enriching their biological functions and main pathways can provide more precise, and technically effective biomarkers for early diagnosis and the treatment of cancer.

## Materials and methods

### Samples and tumor characteristics

This study has been approved by the Mashhad University of Medical Sciences, Mashhad, Iran (approval number: 975011). Patient samples with CRC (N = 6) had a diagnosis determined by colonoscopy and confirmed by an expert pathologist report. Only CRC patients with stages I, II & III disease were included. Excluding criteria were patients with prior CRC, other cancers, a clear family history of adenoma polyposis, inflammatory bowel disease, inherited CRC and patients with incomplete colonoscopy and documentation. Normal samples (N = 6) were individuals who were age and sex-matched and underwent colonoscopy screening with a negative for CRC. Demographic profiles, colonoscopy records, alcohol history, smoking, as well as medical history have all been collected. Anal, rectum, sigmoid, cecum, transverse, descending, and ascending colon were defined as the location of the lesion. The data is presented in Table 1.

**Table 1:** Clinicopathological characteristics of 6 CRC samples matched with normal controls

ID	Age	Sex	Drug	Smoking	Hubble-bubble	Location of tumor	Grade	Result
65T	56	Male	Yes	Yes	No	Cecum	2	Well differentiated Adenocarcinoma
16N	60	Male	Yes	No	No	Cecum	2	
20T	59	Male	No	No	No	Cecum	2	Adenocarcinoma, moderately differentiated
4N	56	Male	No	No	No	Cecum	2	
31T	61	Male	Yes	Yes	No	Sigmoid	2	Adenocarcinoma, moderately differentiated
10N	60	Male	Yes	Yes	No	Sigmoid	2	
35T	71	Male	No	No	No	Rectum	2	Adenocarcinoma, moderately differentiated
7N	74	Male	No	No	No	Rectum	2	
45C	69	Male	No	No	No	Rectum	2	Adenocarcinoma, moderately differentiated
8N	60	Male	No	No	No	Rectum	2	
67C	70	Male	No	No	No	Recto sigmoid	2	Adenocarcinoma, well differentiated
14N	76	Male	No	No	No	Recto sigmoid	2	

### Date processing

#### SureSelectXT methyl-seq data information and DMGs identification

In the process of SureSelectXT Methyl-Seq to detect DMRS, we used DMRFusion tool [14] to normalize the raw data of Agilent SureSelect [15]. In addition to this gene methylation profiling, several other gene methylation profiling datasets (GSE48684, GSE53051, GSE77718, GSE101764, and GSE42752) were obtained from Gene Expression Omnibus (GEO, [16]) of the National Center for Biotechnology Information (NCBI). Data from each methylation was analyzed independently in a GEO series using the online program GEO2R (http://www.ncbi.nlm.nih.gov/geo/geo2r/) to identify differentially methylated genes (DMGs) by contrasting the two CRC and normal mucosa tissue sample groups through setup conditions. The TCGA database was also used to download DNA methylation data (IlIumina Human Methylation 450). Finally, data from 321 tissues (300 colorectal tumor tissues and 21 non-tumor tissues) with DNA methylation information were collected. R 3.5.1 program (https://www.rproject.org/) was applied to evaluate the TCGA results. MethylMix version 3.7 package [17] was used to study the methylation data. These DMGs were compared with our current experiment results [hypo- or hyper-DMRs] in order to detect the robust hypo- or hyper-methylated genes. Only genes which met the cut-off criteria of p value < 0.05 and |log_2_ fold change (FC)|≥ 1.5 were considered as DMGs.

#### Functional and pathway enrichment analysis of DMGs

Gene functional annotation and pathway enrichment analysis is performed using the Database for Annotation, Visualization, and Integrated Discovery (DAVID, https://david.ncifcrf.gov/) [18]. Gene ontology (GO) is a classification system that includes cellular components, molecular functions, and biological processes [19]. Pathway enrichment study using the Kyoto Encyclopedia of Genes and Genomes (KEGG) [20] has been performed for selected DMGs with the thresholds of p value < 0.05 [21, 22].

#### Protein–protein interaction (PPI) network construction and module analysis

PPI research is important to understand the molecular mechanisms of key cellular involvement in carcinogenesis. We generated a PPI network of DMGs using the Search Tool for the Retrieval of Interacting Genes (STRING) database. Interaction score of 0.4 was regarded as the cut-off criterion [23] and the PPI was visualized by Cytoscape software 3.7.2 [24]. Molecular Complex Detection (MCODE) was used to find dense clique-like structures within a network, with MCODE score > 3 and number of nodes ≥ 4. The hub genes were identified on the basis degree and through the value of using cytoHubba [25, 26]. Functional enrichment analysis of the genes in the individual modules was performed by DAVID with a significance level of p < 0.05.

#### Survival analysis

Candidate hub genes were subjected to a survival study to see how they affected CRC survival. The Kaplan Meier plotter was used to perform overall survival (OS) analysis using TCGA methylation results. We divided the patients into two groups based on their Mean. In other words, in the survival study, the groups were split into two categories: low methylation level and high methylation level. The hazard ratio for OS was estimated, and the P value was calculated using log-rank test.

#### Experimental validation in the CCLE database

The methylation levels of key genes were analyzed in other types of cancer cell lines from the TCGA database to equate the function of hub genes in CRC with that of other cancer types from the CCLE database (https://portals.broadinstitute.org/ccle/about).

### Statistical analysis

During statistical evaluation of DMGs, false discovery rate (FDR) was applied for Student t-test with the criteria p < 0.05 in all comparisons. Db-values were used to identify variations in methylation between diagnostic types (the differences of the average b-values of sample groups).

## Results

### Identification of DMGs

A total of 871-shared DMGs (496 hyper and 375 hypo) were obtained in the comparison of the tumor and the normal group, according to the cut-off value selected for the screening. Then, totally 320 genes (215 hyper and 105 hypo) were detected as common genes between our patients and the selected GEO and TCGA datasets.

### Gene ontology enrichment analyses

**Fig. 1:**
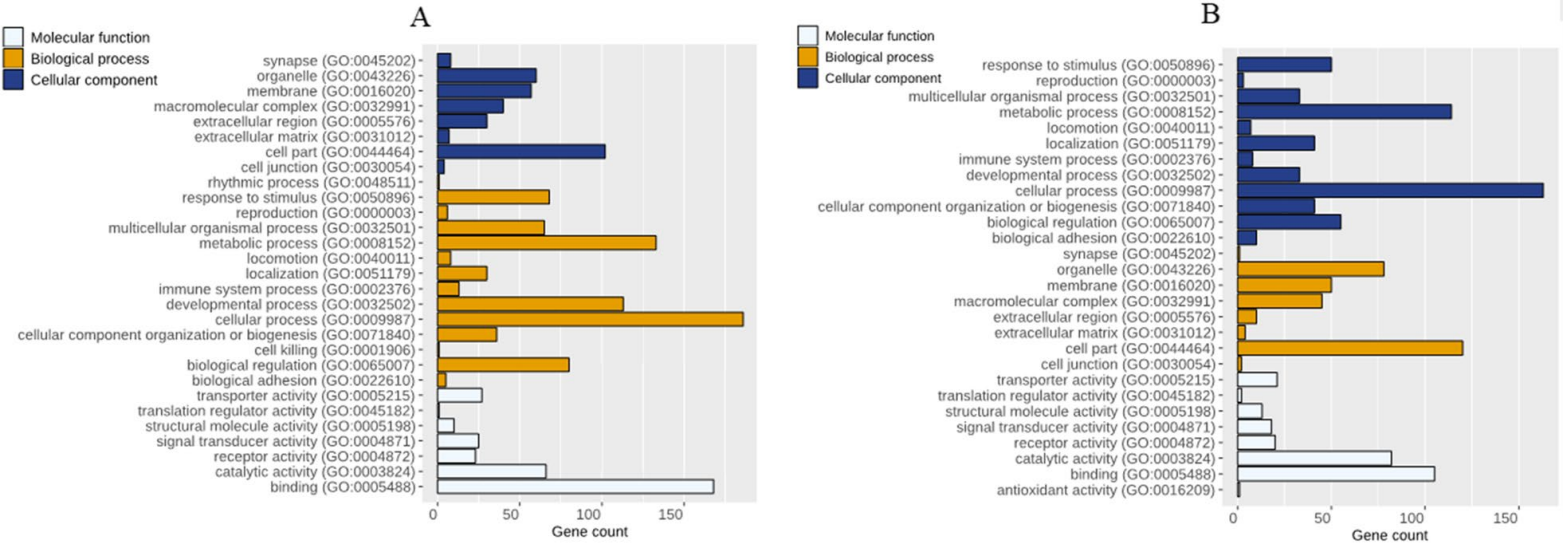
GO-Enrichment Analysis of **A** hyper-methylated genes and **B** hypo-methylated genes

In view of the analysis in DAVID for hyper-DMRS in CRC (Fig. 1), gene ontology enrichment analysis demonstrated biological process (BP) including biological adhesion, cell killing, biological regulation, cellular component organization or biogenesis, developmental process, immune system process, metabolic process, localization, multicellular organismal process, cellular process, reproduction, rhythmic process and response to stimulus. As molecular function (MF), enrichment analysis indicated histone and protein binding, catalytic activity, structural molecule activity, signal transducer, translation regulator activity and transporter receptor activity sites. Besides, for cell component (CC) enrichment analysis displayed apparatuses cell membrane, synapse, organelle, in which DMGs might play a critical role.

In view of the analysis in DAVID for hypo-DMRS in CRC (Fig. 1), GO enrichment analysis demonstrated BP including biological regulation, biological adhesion, cellular component organization or biogenesis, cellular process, developmental process, localization, metabolic process, locomotion, and multicellular organismal process. As MF, enrichment analysis indicated histone and protein binding, catalytic activity, structural molecule activity, signal transducer, translation regulator activity and transporter receptor activity sites. Besides, for cell component enrichment analysis displayed cell junction, cell part, extracellular matrix, extracellular region, macromolecular complex, membrane, organelle and synapse.

### KEGG pathway analysis

To investigate the potential mechanism of methylation-regulated genes in CRC, pathway enrichment analysis was used. KEGG pathway enrichment analysis suggested that the hyper-DMGs were significantly enriched in 10 pathways including: Wnt signaling pathway, hedgehog signaling pathway, basal cell carcinoma, pathways in cancer, melanogenesis, proteoglycans in cancer, hippo signaling pathway, transcriptional misregulation in cancer, prostate cancer and calcium signaling pathway (Table2).

**Table 2:** KEGG pathway analysis of the hyper-DMGs

ID	Pathway description	Gene count	False discovery rate	Matching proteins in network (labels)
*5217*	Basal cell carcinoma	12	5.79E−07	BMP2,FZD1,GLI3,TCF7,TCF7L1,WNT1,WNT16,WNT2,WNT3A,WNT5A,WNT6,WNT7B
*4340*	Hedgehog signaling	11	1.25E−06	BMP2,GAS1,GLI3,IHH,WNT1,WNT16,WNT2,WNT3A,WNT5A,WNT6,WNT7B
*4340*	Hedgehog signaling	11	1.25E−06	CTBP2,DKK1,FZD1,NFATC1,SFRP2,SFRP5,TCF7,TCF7L1,WNT1,WNT16,WNT2,WNT3A,WNT5A,WNT6,WNT7B
*4310*	Wnt signaling	15	2.78E−05	CREB3L1,FZD1,GNAS,TCF7,TCF7L1,WNT1,WNT16,WNT2,WNT3A,WNT5A,WNT6,WNT7B
*4916*	Melanogenesis	12	9.14E−05	BCL2,BMP2,COL4A2,CTBP2,FGF12,FGF3,FGFR1,FZD1,GLI3,IGF1R,JUP,PDGFRA,TCF7,TCF7L1,TGFB1,WNT1,WNT16,WNT2,WNT3A,WNT5A,WNT6,WNT7B
*5200*	Pathways in cancer	22	0.00015	BMP2,FZD1,PPP2R2A,STK3,TCF7,TCF7L1,TGFB1,WNT1,WNT16,WNT2,WNT3A,WNT5A,WNT6,WNT7B
*4390*	Hippo signaling	14	0.000203	CAV1,FGF12,FGF3,FGFR1,FZD1,IGF1R,IGF2,SDC2,TGFB1,TWIST2,WNT1,WNT16,WNT2,WNT3A,WNT5A,WNT6,WNT7B
*5205*	Proteoglycans in cancer	17	0.000286	CDK14,IGF1R,IGFBP3,JUP,MEIS1,NGFR,NR4A3,PAX7,SIX1,TLX3,WNT16,WT1
*5202*	Transcriptional misregulation in cancer	12	0.01	BCL2,CREB3L1,CREB5,FGFR1,IGF1R,PDGFRA,TCF7,TCF7L1
*5215*	Prostate cancer	8	0.0177	AVPR1A,CACNA1I,CD38,GNAS,GRIN2A,GRIN2C,ITPKB,NOS1,PDE1B,PDGFRA,TACR1
*4020*	Calcium signaling	11	0.0474	CTBP2,DKK1,FZD1,NFATC1,SFRP2,SFRP5,TCF7,TCF7L1,WNT1,WNT16,WNT2,WNT3A,WNT5A,WNT6,WNT7B
*4340*	Hedgehog signaling	11	1.25E−06	CREB3L1,FZD1,GNAS,TCF7,TCF7L1,WNT1,WNT16,WNT2,WNT3A,WNT5A,WNT6,WNT7B

**Fig. 2:**
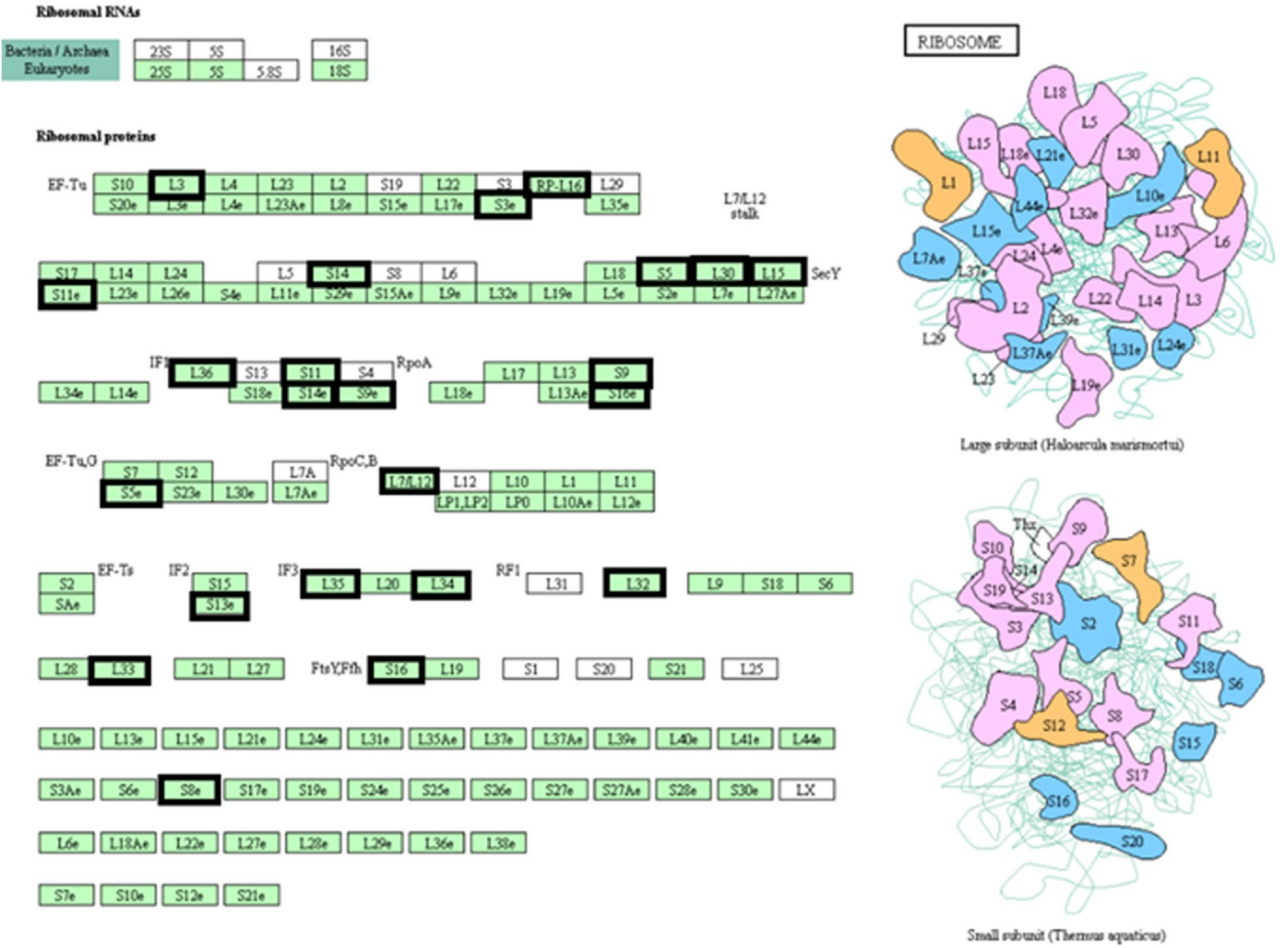
Ribosome pathway and matching genes in network. Our founded genes are marked by a bold box in the pathway

Interestingly, KEGG pathway analysis suggested that the hypo-DMGs were enriched in only one pathway termed “Ribosome with pathway” (Fig. 2).

### PPI network construction

**Fig. 3:**
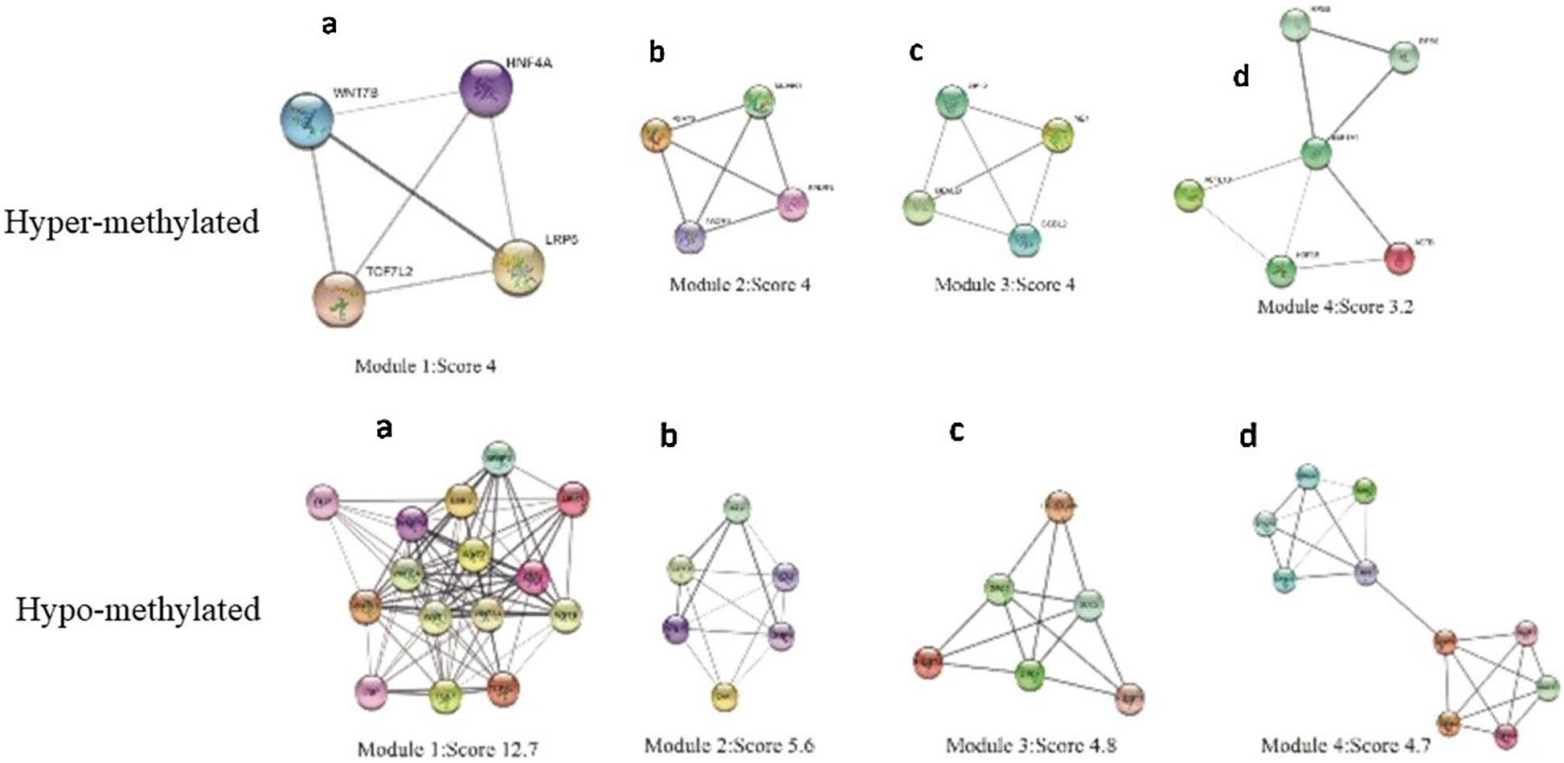
Top four modules of hyper and hypo-methylated genes; **a**–**d** 4 top modules

A total of 215 PPI nodes of the hyper-DMGs were constructed on the basis of STRING database (Fig. 3 and Additional file 2: Figure S2). A total of 105 PPI nodes of the hypo-DMGs were constructed through the basis of STRING database (Fig. 3 and Additional file 3: Figure S3).

**Fig. 4:**
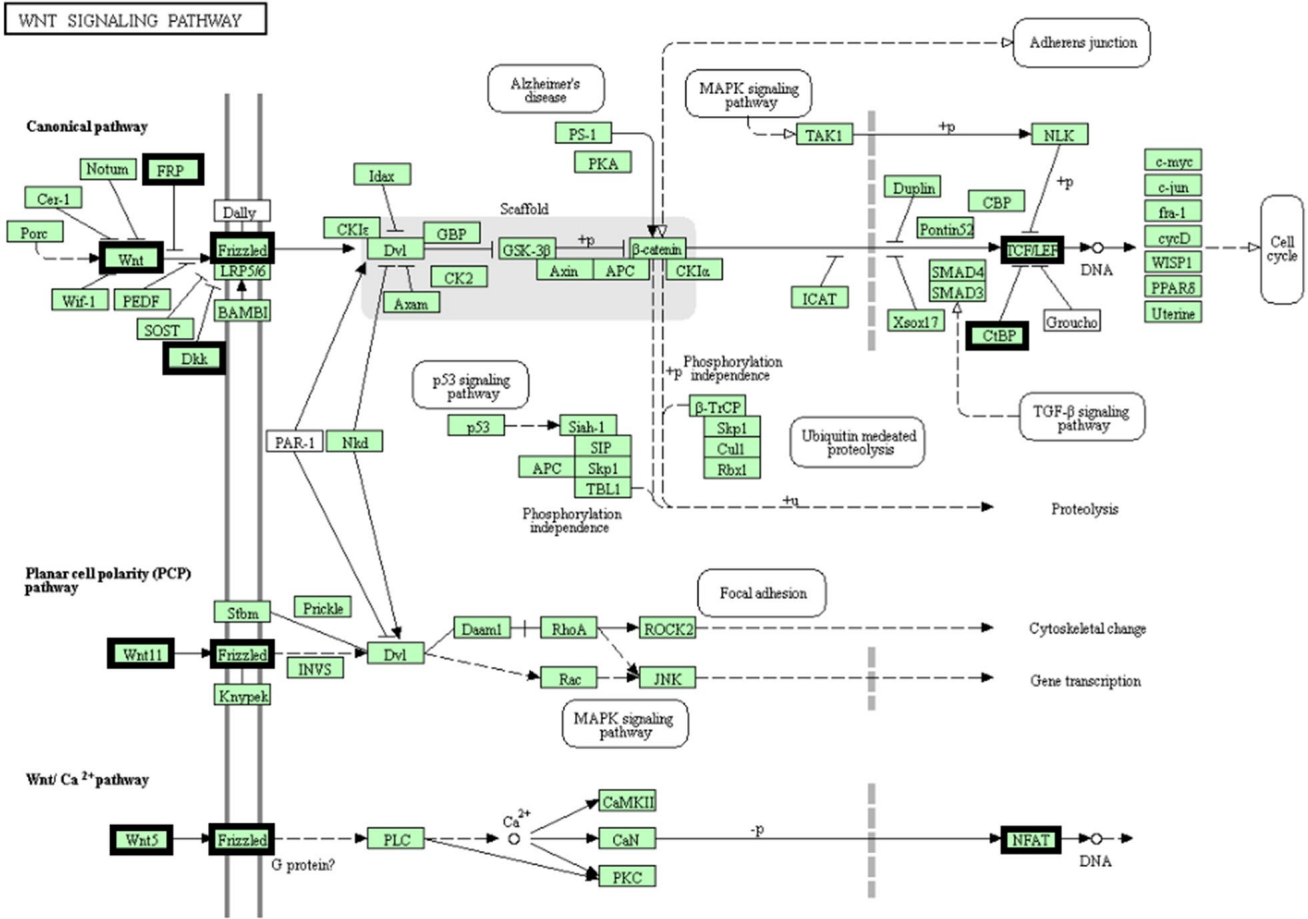
Wnt signaling pathway and matching genes in network. Our founded genes are marked by a bold box in the pathway

The four node proteins, including *WNT2*, *SFRP2*, *ZNF726* and *BMP2* which showed a close interaction with other node proteins, were chosen as hub proteins. One important module was selected, based on the number of nodes > 4 as shown in Additional file 2: Figure S2.The key module demonstrated functions enriched in pathways such as Wnt signaling (Table 2 and Fig. 4). PPI network of DMGs illustrated the overview of the functional connections, in which the top 4 hub genes were presented as *WNT2*, *SFRP2*, *ZNF726* and *BMP2*.

Furthermore, Wnt signaling pathway as a crucial pathway in tumorigenesis was unveiled by KEGG enrichment analysis. The aberrant activation of canonical Wnt signaling is a hallmark occurrence for colorectal carcinogenesis.

### Survival analysis of candidate hub genes

**Fig. 5:**
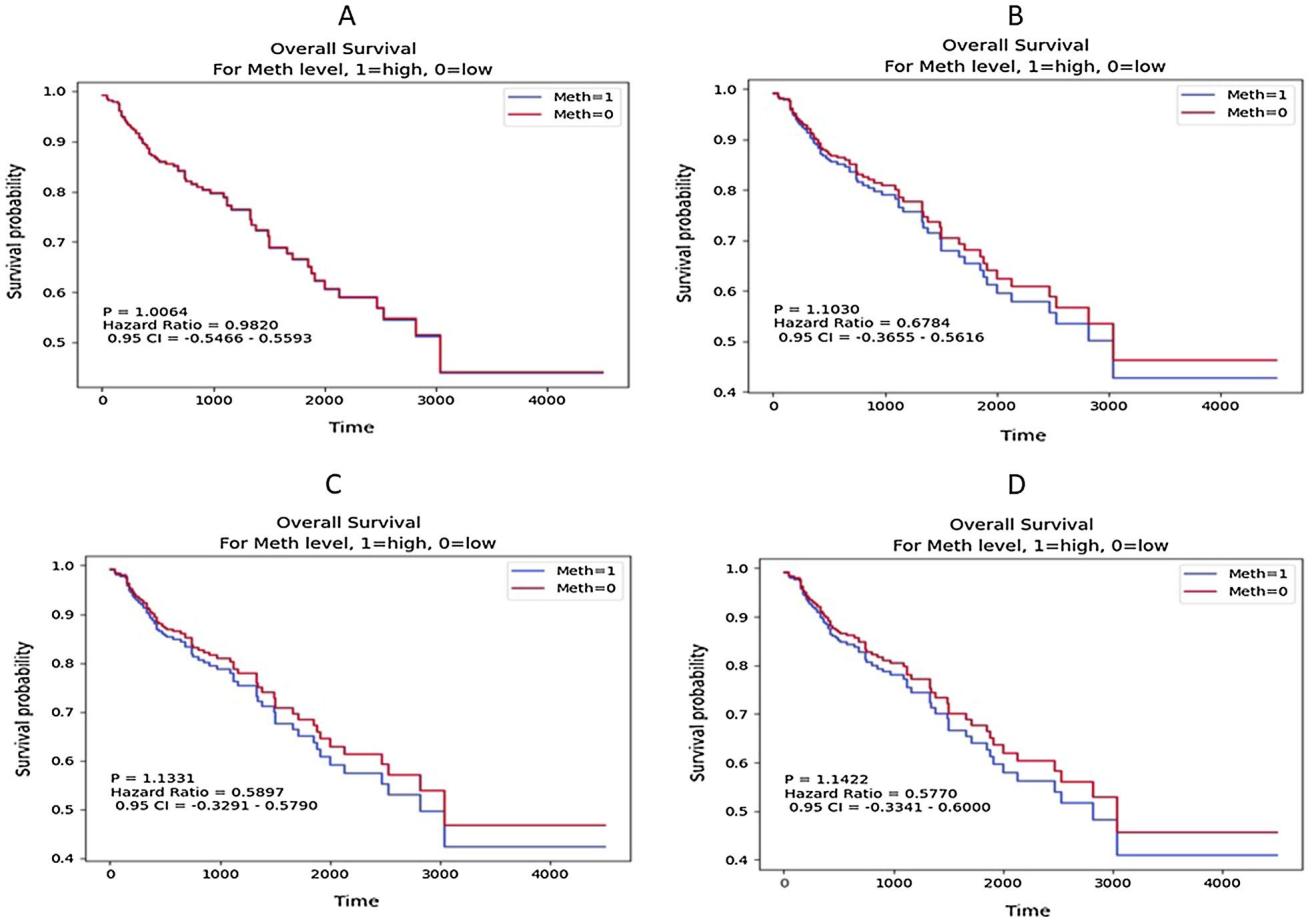
The survival analysis of four hub genes by the KM-plotter. **a** *WNT2*; **b** *SFRP2*; **c** *ZNF726*; **d** *BMP2*

In our research, associations between methylation of candidate hub genes and OS of CRC patients were analyzed using the KM approach to estimate the hub genes' prognostic significance. The results indicated that high methylation of *SFRP2*, *ZNF726* and *BMP2* lead to lower OS rate than low methylation. On the other hand, *WNT2* gene methylation were not significantly relevant to OS (Fig. 5).

### Experimental validation in cancer cell lines

The methylation of hub genes were further examined in various types of tumor cells. As presented in Additional file 4: Figure S4, among other tumor cell lines, *WNT2* and *ZNF726* had the highest methylation in CRC, indicating that the role of *WNT2* and *ZNF726* genes in the CRC may be linked to methylation regulation.

## Discussion

A large amount of core slice data has been created by the widespread use of gene methylation, with the majority of the data being deposited and processed in public databases. Integrating and re-analyzing these data will yield important insights for future analysis. In recent years, several methylation sequencing data profiling experiments on CRC have been conducted [27] and hundreds of DMGs have been obtained. However, the findings are often limited or contradictory due to the variability of the tissue or sample in independent studies or the findings of a single cohort study.

In this study, the DMGs from CRC patients and normal participants were screened. From the Agilent SureSelect analysis, eight hundred and seventy-one DMGs (496 hyper and 375 hyper) were classified with selecting parameters of p value < 0.05 and |log2 fold change (FC)|≥ 1.5.

In view of the analysis in DAVID for DMRS in CRC, GO enrichment analysis demonstrated BP including biological regulation, biological adhesion, cellular component organization or biogenesis, cellular process, developmental process, localization, metabolic process, locomotion, and multicellular organismal process. It has been reported that cell proliferation and lack of cell adhesion are a common characteristic of cancers, particularly in CRC [28]. Previous research studies have showed that the cell cycle plays a critical role in cancer development by controlling cell division and specifically in CRC the incidence and metastasis are significantly associated with cell proliferation, cell cycle deregulation, and cell cycle related kinase [29].

PPI network of DMGs illustrated the overview of the functional connections, in which the top 4 hub genes were presented as *WNT2*, *SFRP2*, *ZNF726* and *BMP2*.

KEGG enrichment analysis has revealed the Wnt signaling pathway as a critical pathway in tumorigenesis which is characterized by irregular activation of canonical Wnt signaling [30, 31].

The most important modules were extracted from MCODE and 14 central node genes were identified as the majority of the corresponding genes were found to be involved in cellular process and protein binding. Hub genes including *WNT2*, *BMP2*, *ZNF726*, *and SFRP2* were suggested as potentially diagnostic and therapeutic targets for CRC.

Secreted Frizzled Related Protein 2 (*SFRP2*) gene encode extracellular Wnt signaling inhibitors that are often inhibited by CRC promoter methylation [32]. In several tumors, including CRC, both of these methylation events have been identified as prognostic indicators of patient outcome and tumor subtype. *SFRP2* gene is found in the upstream of the canonical Wnt signaling pathway which its methylation contributes to the down-regulation of gene expression, inhibition of gene action, activation of the Wnt pathway and promotion of CRC [33, 34]. The DNA hyper-methylation of this gene may be used as a biomarker for CRC detection [35].

Bone morphogenetic proteins (BMPs) belong to the transforming growth factor beta superfamily (TGFβ) and are essential regulators of embryogenesis and body axes. Adult tissue homeostasis is controlled by regulating cell production, apoptosis and differentiation [36]. *BMP2* has been identified as a tumor suppressor gene in CRC. Nevertheless, the association between *BMP2* and clinicalopathological influences has not been established in clinical CRC cases [37].

ZNF proteins, such as ZNF346, ZNF638, and ZNF768, are used as capture antigens in CRC to detect autoantibodies [38]. Furthermore, in a previous study, epigenetically activated ZNF726 was identified as a prognosis‑associated gene for CRC [39].

The important GO concepts were linked to positive regulation of gene expression, protein binding, and cellular protein metabolic mechanism, as indicated by DAVID study. Staring at the signaling pathway, we noticed that the Wnt signaling pathway, the Transcriptional Cancer Misregulation, and the Hedgehog signaling pathway were highly enriched which been previously reported [36].

In the present study, some limitations should be mentioned. Although the number of patients who were analyzed in this study was small, our results were confirmed by the results of analyzing data taken from other databases. However, larger sample size could lead to more powerful results. Besides, molecular studies should also be conducted to further validate the findings of our investigation. Hub genes including *WNT2*, *SFRP2*, *ZNF726* and *BMP2* were suggested as potentially diagnostic and therapeutic targets for CRC. Targeting the identified pathways particularly WNT signaling could close us to a more efficient treatment for CRC. More clinical and biological experimental evidence on the candidate genes is needed to confirm their clinical utility, which would help clinicians establish new diagnostic and therapeutic strategies for patients with CRC.

## Conclusions

The results obtained using an integrated bioinformatics framework allowed to identify DMG candidate genes and pathways in CRC, which could enhance our understanding of the consequences and also the underlying molecular events in cancer initiation and progression. In addition, some of which following validation could be used for clinical applications.

## Supplementary Information

**Additional file 1: Figure S1.** Project workflow.

**Additional file 2: Figure S2.** PPI network of hyper-methylated genes.

**Additional file 3: Figure S3.** PPI network of hypo-methylated genes.

**Additional file 4: Figure S4.** Verification of DNA‑methylation of four hub genes in other tumor and colorectal cell lines.

## Data Availability

All data used are publicly available.
