# Supplementary figures and images for: Aberrantly methylated-differentially genes and pathways among Iranian patients with colorectal cancer
Source: Cancer Cell Int. 2021 Jul 3;21:346. doi: 10.1186/s12935-021-02053-0 (PMC8255023; doi:10.1186/s12935-021-02053-0)

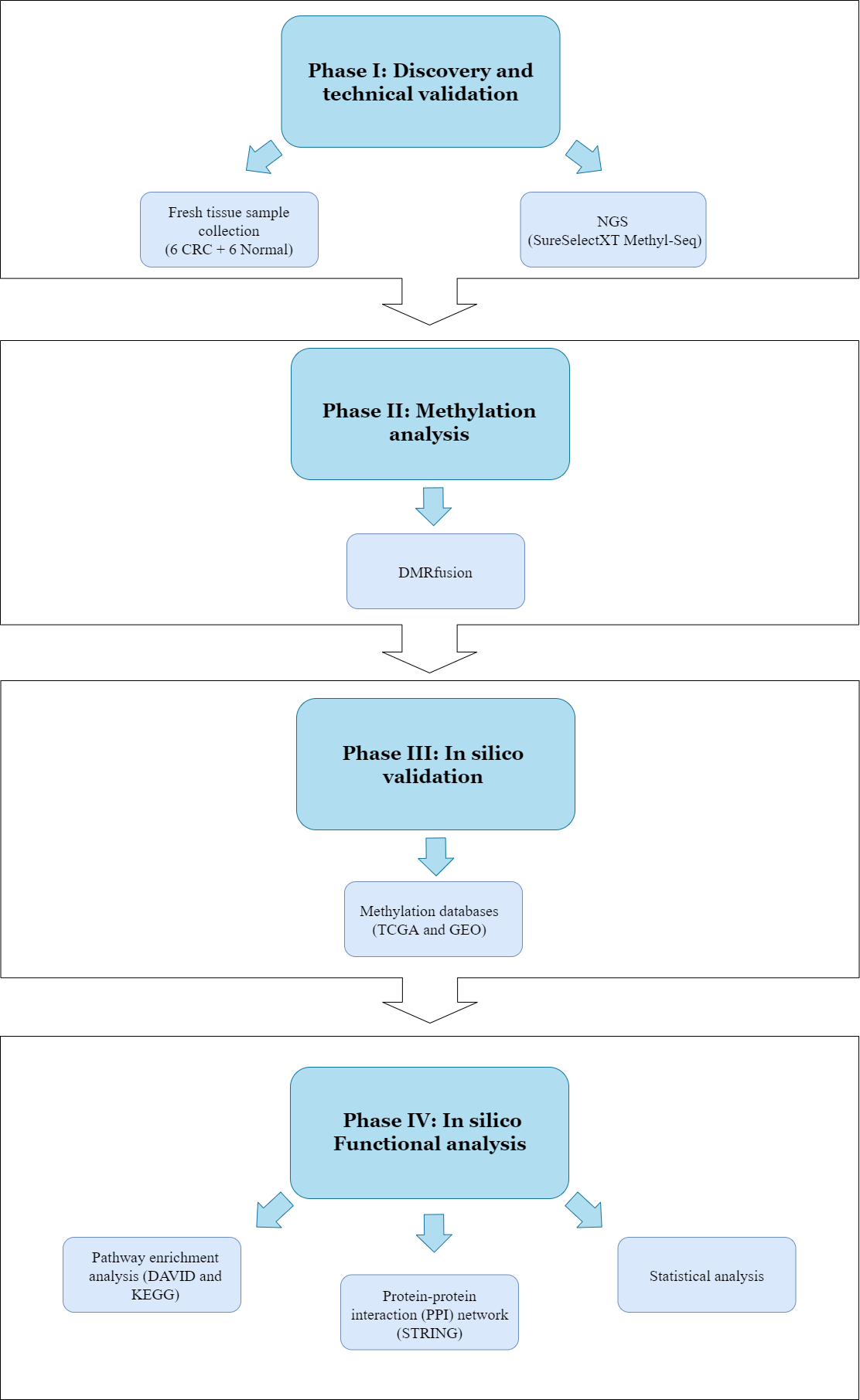

Supplement: Supplementary file 1 — Additional file 1: Figure S1. Project workflow. [file 12935_2021_2053_MOESM1_ESM.png]

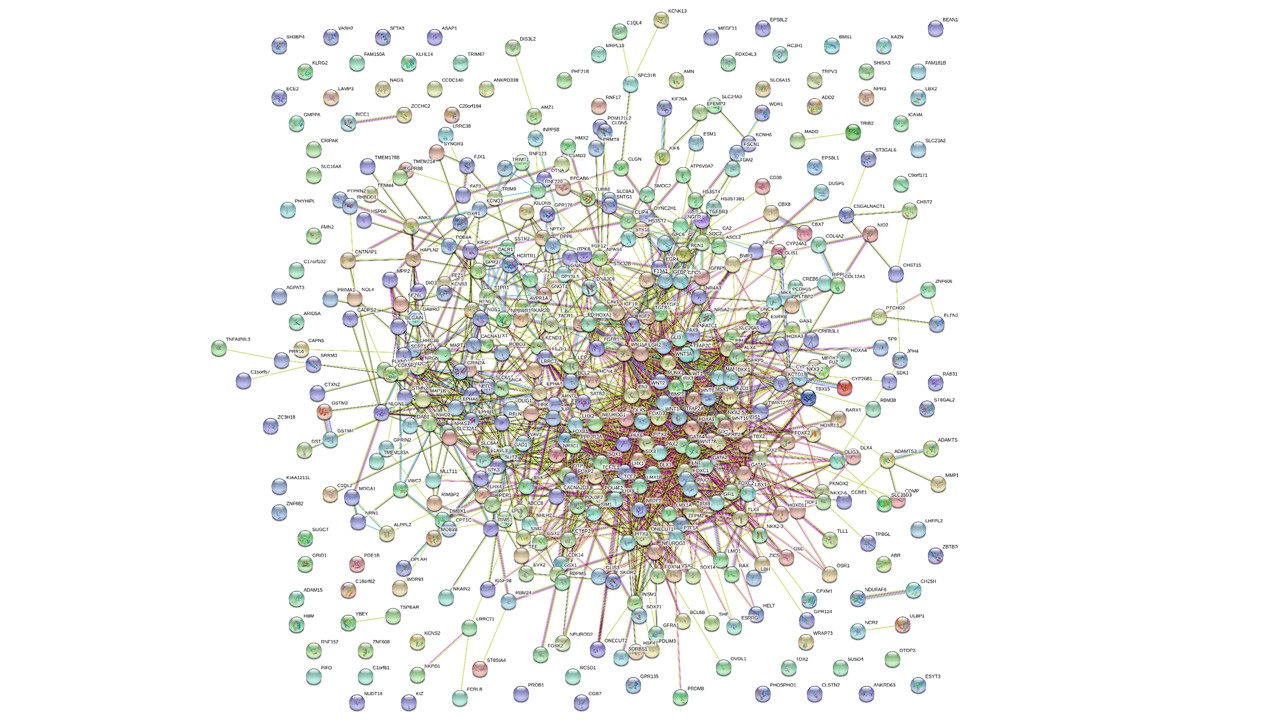

Supplement: Supplementary file 2 — Additional file 2: Figure S2. PPI network of hyper-methylated genes. [file 12935_2021_2053_MOESM2_ESM.jpg]

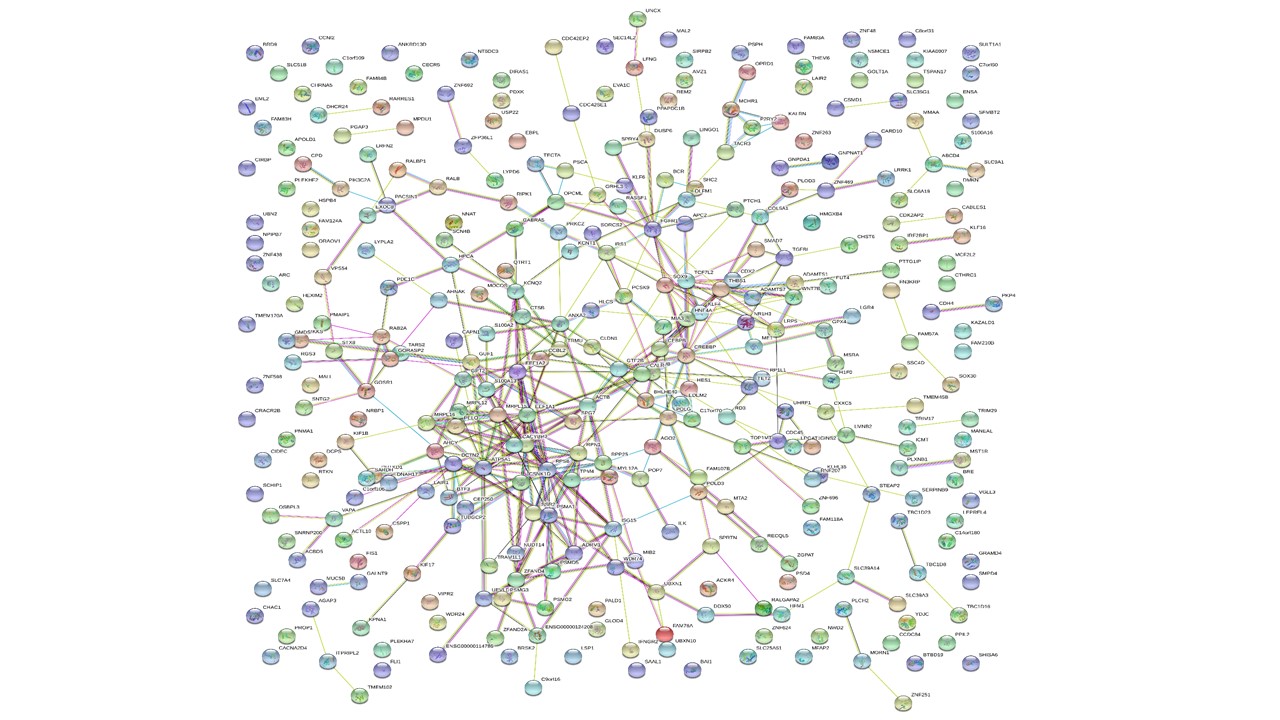

Supplement: Supplementary file 3 — Additional file 3: Figure S3. PPI network of hypo-methylated genes. [file 12935_2021_2053_MOESM3_ESM.jpg]

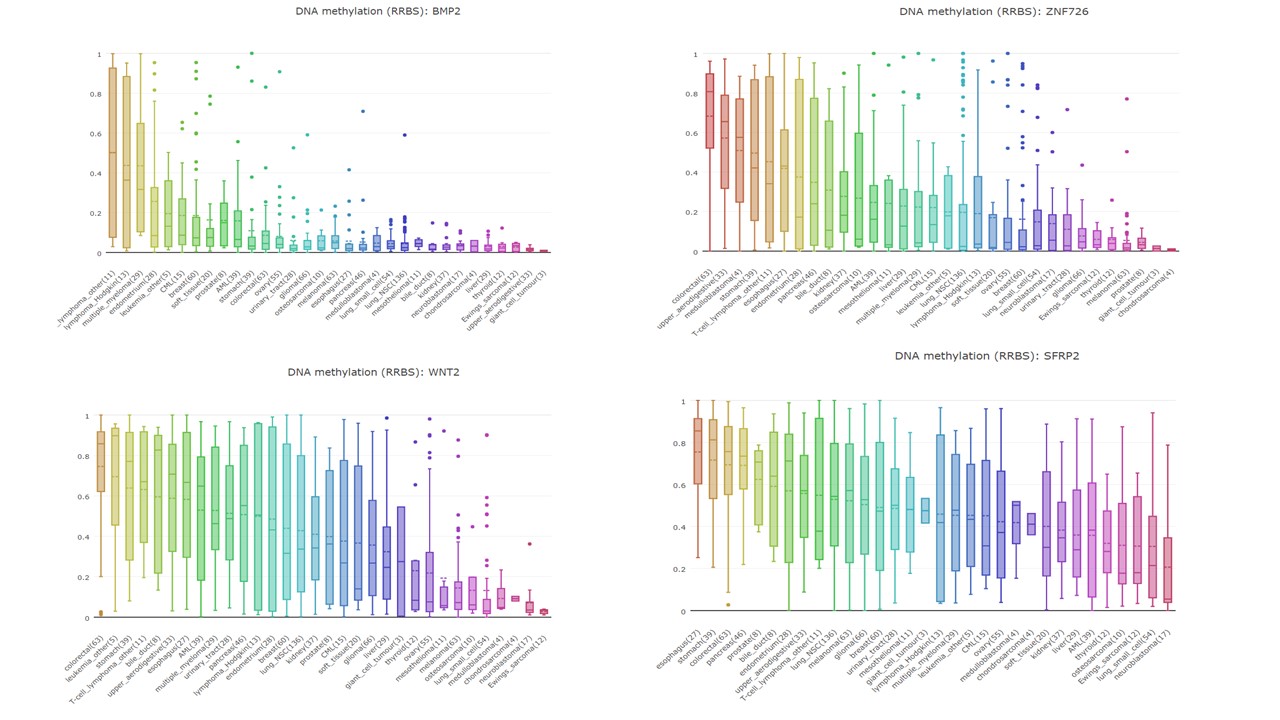

Supplement: Supplementary file 4 — Additional file 4: Figure S4. Verification of DNA‑methylation of four hub genes in other tumor and colorectal cell lines. [file 12935_2021_2053_MOESM4_ESM.jpg]
